# *β*_*2*_*microglobulin* mRNA expression levels are prognostic for lymph node metastasis in colorectal cancer patients

**DOI:** 10.1038/sj.bjc.6604399

**Published:** 2008-05-27

**Authors:** J Shrout, M Yousefzadeh, A Dodd, K Kirven, C Blum, A Graham, K Benjamin, R Hoda, M Krishna, M Romano, M Wallace, E Garrett-Mayer, M Mitas

**Affiliations:** 1 College of Health Professions-Cytology and Biosciences, Medical University of South Carolina, Charleston, SC, USA; 2 Department of Surgery, Medical University of South Carolina, Charleston, SC, USA; 3 Department of Pathology and Laboratory Medicine, Medical University of South Carolina, Charleston, SC, USA; 4 Department of Pathology, Mayo Clinic College of Medicine, Jacksonville, FL, USA; 5 Department of Medicine, Mayo Clinic College of Medicine, Jacksonville, FL, USA; 6 Department of Biostatistics, Bioinformatics, and Epidemiology, Medical University of South Carolina, Charleston, SC, USA

**Keywords:** formalin-fixed paraffin-embedded; gene expression; prognostic assay, colorectal cancer, real-time PCR, area under the curve

## Abstract

Colorectal cancer (CRC) is the fourth most common non-cutaneous malignancy in the United States and the second most frequent cause of cancer-related death. One of the most important determinants of CRC survival is lymph node metastasis. To determine whether molecular markers might be prognostic for lymph node metastases, we measured by quantitative real-time RT–PCR the expression levels of 15 cancer-associated genes in formalin-fixed paraffin-embedded primary tissues derived from stage I–IV CRC patients with (*n*=20) and without (*n*=18) nodal metastases. Using the mean of the 15 genes as an internal reference control, we observed that low expression of *β*_*2*_*microglobulin* (*B2M*) was a strong prognostic indicator of lymph node metastases (area under the curve (AUC)=0.85; 95% confidence interval (CI)=0.69–0.94). We also observed that the expression ratio of *B2M/Spint2* had the highest prognostic accuracy (AUC=0.87; 95% CI=0.71–0.96) of all potential two-gene combinations. Expression values of *Spint2* correlated with the mean of the entire gene set at an *R*^2^ value of 0.97, providing evidence that *Spint2* serves not as an independent prognostic gene, but rather as a reliable reference control gene. These studies are the first to demonstrate a prognostic role of *B2M* at the mRNA level and suggest that low *B2M* expression levels might be useful for identifying patients with lymph node metastasis and/or poor survival.

Colorectal cancer (CRC) is the fourth most common noncutaneous malignancy in the United States and the second most frequent cause of cancer-related death. In 2007, an estimated 153 760 cases of CRC will be diagnosed, and 52 180 people will die from the disease (Jemal *et al*, 2006). The most important determinant of colon cancer survival is stage. The tumour–node–metastasis system, as defined by the American Joint Committee on Cancer, is the most commonly used cancer staging system and classifies colon cancer into four stages based on the depth of invasion of the bowel wall (T), extent of regional lymph node involvement (N), and presence of distant sites of metastatic disease (M) (Greene *et al*, 2002). Stage I includes T1 and T2 tumours without nodal or distant metastases and most patients with this disease will be cured with segmental colectomy alone. The overall 5-year survival (OS) of this stage is 93.2%. Stage II is subdivided into two classes (IIA and IIB; OS=84.7 and 72.2%, respectively) and includes T3 and T4 tumours, respectively. Like stage I, nodal or distant metastasis is absent in stage II disease. Although many patients with stage II disease will be cured by surgical resection alone, many patients with completely resected stage II disease will ultimately die from colon cancer (Jemal *et al*, 2006). Stage III disease includes tumours that do contain nodal disease but do not contain distant metastases. After complete surgical resection, these patients face a 50–60% chance of developing recurrent disease. A survival benefit from adjuvant 5-fluorouracil-based chemotherapy has been firmly established in these patients, and recent data have shown further efficacy through the inclusion of oxaliplatin into adjuvant treatment programmes (Chung and Saltz, 2007; Wolpin *et al*, 2007).

At present, the standard procedure for determining the spread of metastatic disease to lymph nodes is pathological examination of ∼20 resected lymph nodes stained with haematoxylin and eosin (H&E). We reasoned that an assay that was able to identify patients with metastatic disease by measuring RNA expression levels of select genes would be helpful for making clinical decisions. In the current study, we investigated whether expression levels of 14 cancer-associated genes in the primary tumour were correlated with lymph node metastases. These 14 genes are derived from a set of 22 that our laboratory has previously identified as being overexpressed in various cancers (Reed *et al*, 2007).

In addition to the set of 14 genes, we also chose to examine the expression of *β*_*2*_*microglobulin* (*B2M*). Our rationale for the inclusion of *B2M* was based on the following observations: (1) 32% of *B2M*^null^ × IL-2^null^ mice develop adenocarcinoma in the proximal half of the colon between 6 and 12 months (Shah *et al*, 1998), (2) out of 19K genes screened from 25 matched CRC tissue and normal mucosa, *B2M* was the most highly downregulated gene in CRC (Bianchini *et al*, 2006), (3) oncogenic K-ras mutations (which are present in the majority of CRC) inhibit the expression of *B2M* and other interferon (IFN)-responsive genes (Klampfer *et al*, 2003), (4) downregulation of *B2M* in CRC has been confirmed by real-time RT–PCR (Bianchini *et al*, 2006), and (5) the level of expression of *B2M* is very high and can be reliably measured in formalin-fixed paraffin-embedded (FFPE) tissues (Chen *et al*, 2007).

## MATERIALS AND METHODS

### Patients and tissues

This study was approved by the Institutional Review Boards at the Medical University of South Carolina and by the Mayo Clinic College of Medicine in Jacksonville. *Metastatic and benign lymph nodes from colon cancer patients.* Medical records were searched for patients who underwent surgical resection and who did (*n*=7) and did not (*n*=7) have associated lymph node metastases at the Mayo Clinic. A 50-*μ*m-thick section was cut for real-time RT–PCR studies and a 5-*μ*m-thick section was used for H&E staining. The presence of metastatic disease in lymph nodes identified as positive was confirmed by the study pathologist. *Primary tumour specimens.* Medical records were searched for colon cancer patients who had at least one metastatic lymph node (*n*=20) or no metastatic lymph nodes (*n*=18). Duplicate 50-*μ*m-thick sections were cut for real-time RT–PCR studies and a 5-*μ*m-thick section was used for H&E staining.

### RNA isolation from paraffin sections

mRNA extraction followed the method of Specht *et al* (2001). Briefly, paraffin-embedded tissue sections were deparaffinised twice with 1 ml of xylene at 37°C or room temperature for 10 min. The pellet was subsequently washed with 1 ml of 100, 90, and 70% of ethanol and air-dried at room temperature for 2 h. The pellet was resuspended in 200 *μ*l of RNA lysis buffer (2% lauryl sulphate, 10 mmol l^−1^ Tris-HCl (pH 8.0), and 0.1 mmol l^−1^ EDTA) and 100 *μ*g of proteinase K and incubated at 60°C for 16 h. RNA was extracted using 1 ml of phenol/chloroform (5 : 1) solution (Sigma, St Louis, MO, USA). The aqueous layer containing RNA was transferred to a new 1.5 ml tube. Phenol/chloroform extraction was performed a total of three times. RNA was precipitated with an equal volume of isopropanol, 0.1 volume of 3 mol l^−1^ sodium acetate, and 100 *μ*g of glycogen at −20°C for 16 h. After centrifugation at 12 000 r.p.m. for 15 min (4°C), the RNA pellet was washed with 70% of ethanol and air-dried at room temperature for 2 h. Finally, the pellet was dissolved in 12 *μ*l of DEPC water and treated with DNase before complementary DNA (cDNA) synthesis as described in the text.

### Complementary DNA synthesis and real-time RT–PCR

Complementary DNA was made from 6 *μ*l of RNA described above, 200 U of M-MLV reverse transcriptase (Promega, Madison, WI, USA), and a panel of truncated gene-specific primers (Table 1). Real-time RT–PCR was performed on a PE Biosystems Gene Amp® 7300 or 7500 Sequence Detection System (Foster City, CA, USA). With the exception of the SYBR Green I master mix (purchased from Qiagen, Valencia, CA, USA), all reaction components were purchased from PE Biosystems. Standard reaction volume was 10 *μ*l and contained 1 × SYBR RT–PCR buffer, 3 mM MgCl_2_, 0.2 mM each of dATP, dCTP, and dGTP, 0.4 mM dUTP, 0.1 U UNG Erase enzyme, 0.25 U AmpliTaq Gold, 0.35 *μ*l cDNA template, and 50 nM of oligonucleotide primer. Initial steps of RT–PCR were 2 min at 50°C for UNG Erase activation, followed by a 10 min hold at 95°C. Cycles (*n*=40) consisted of a 15 s melt at 95°C, followed by a 1 min annealing/extension at 60°C. The final step was a 60°C incubation for 1 min. All reactions were performed in triplicate. Before cDNA synthesis, RNA was treated with or without DNase as described in the text.

### Gene expression and statistical analysis

To quantitate gene expression, the Δ*C*_t_ method was used. As an internal reference, we used either the mean *C*_t_ value of all genes or the median value as described in the text. A primary tumour sample was considered to have sufficient mRNA if its mean *C*_t_ value was <35.2 (38 out of 38 samples; mean±s.d. of all samples=27.5±4.06). Area under the curve (AUC) measurements were performed for single gene analysis using MedCalc software (Mariakerke, Belgium); patients were dichotomised according to lymph node metastasis status. For AUC analysis of *B2M/gene X* expression ratios, Δ*C*_t_ values of 14 different gene combinations were obtained by subtracting the *C*_t_ value of *B2M* from the other genes. Area under the curve analysis was then performed using MedCalc software. Associations between categorical values were assessed using Fisher's exact test. For ordinal variables (e.g. T-stage, pathologic stage), we also used *t*-test to compare mean levels across lymph node and expression categories due to concern over sparseness. Correlation coefficient analysis of potential reference genes was performed using Microsoft Excel software.

## RESULTS

**Figure 1:**
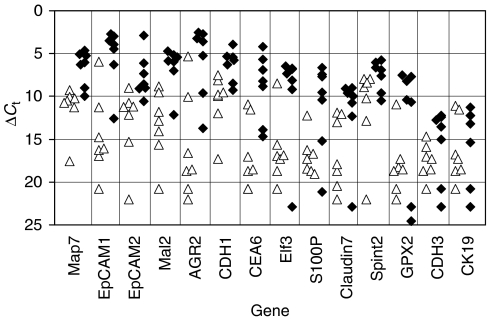
Real-time RT–PCR analysis of metastatic and benign lymph nodes from colon cancer patients. Real-time PCR analyses of seven benign lymph nodes (left side of each matched data set; open triangles) and seven metastatic lymph nodes (right side of each matched data set; closed diamonds) were performed as described in Materials and Methods using primer pairs for the indicated genes. *C*_t_ values for each gene were determined from triplicate reactions. Δ*C*_t_ values were obtained by subtracting the mean *C*_t_ value of *B2M* (which is highly expressed in normal lymph node tissue) from the mean *C*_t_ value for each respective gene. Note: The mean *B2M C*_t_ value of the metastatic lymph nodes was slightly lower (i.e. *B2M* gene expression was slightly higher) but not significantly different from that of benign tissue (17.8±2.1 *vs* 20.2±2.1, respectively).

Using a novel microarray/bioinformatics approach, previously we identified a set of 22 genes that were predicted to be overexpressed in multiple cancers (Reed *et al*, 2007). To investigate whether these genes were overexpressed in metastatic CRC, we selected 14 genes and measured their level of expression in lymph nodes obtained from CRC patients who were positive (*n*=7) and negative (*n*=7) for metastatic disease by H&E staining. We next performed AUC analysis, the most commonly used statistical method for determining accuracies of diagnostic tests (Henderson, 1993). Receiver–operator characteristic (ROC) curve analysis is based on a plot of sensitivity as a function of 1−specificity. The area under the ROC curve (*W*) is a measure of diagnostic (or prognostic; see below) accuracy such that values between 0.5 and 0.7 indicate low accuracy, values between 0.7 and 0.9 indicate moderate accuracy, and values greater than 0.9 indicate high accuracy (Swets, 1988). We observed that the AUC values for detection of metastatic disease of 11 out of 14 (79%) genes were greater than 0.80 (Table 2 and Figure 1). We conclude from this experiment that the set of 14 genes is highly overexpressed in metastatic CRC and hypothesise that one or more genes in this set may be prognostic for lymph node metastases.

To determine whether expression levels of molecular markers might correlate with lymph node metastases, RNA was isolated from FFPE primary tumour sections as described in Materials and Methods and analysed for the expression of the 14 cancer-associated genes listed in Table 1. For reasons stated in the introduction section, we also included in our marker panel the *B2M* gene. Characteristics of the patients with (*n*=20) and without (*n*=18) lymph node metastases are shown in Table 3. As anticipated, we observed a significant association between lymph node metastases and T-stage, pathologic stage, and tissue differentiation (continuous).

**Figure 2:**
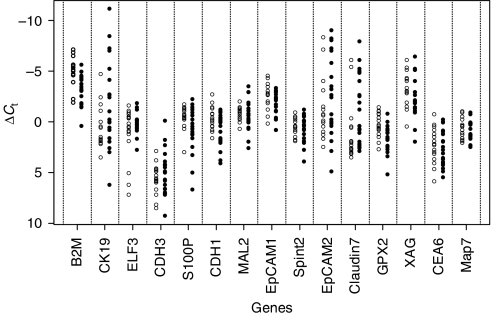
Real-time PCR analyses of FFPE primary tissue from CRC patients. Real-time RT–PCR was performed on patients who did (*n*=18; right side of each matched data set; filled circles) and who did not have (*n*=18; left side of each matched data set; open circles) metastatic lymph nodes from CRC as described in Materials and Methods using primer pairs for the indicated genes. *C*_t_ values for each gene were determined from triplicate reactions.

To evaluate potential prognostic values of the genes, we simply used as an internal reference the mean *C*_t_ value of all 15 genes (Figure 2). Our rationale for this approach was two-fold. First, an ideal internal reference gene in cancer prognostics is one that provides an accurate measure of the amount of *tumour* and not *tissue*. As our genes were selected on the basis of overexpression in tumour and/or metastatic disease, we reasoned that their mean *C*_t_ value should be a reliable measure of tumour content. Second, the number of genes we used for reference was 15, a number sufficiently high to avoid potential problems caused by outliers. In addition to the analysis using the mean *C*_t_ value of the 15-gene set as an internal reference, a separate analysis was also performed using the median *C*_t_ value.

Using the mean of the entire 15-gene set as an internal reference control, we calculated Δ*C*_t_ values for all genes and performed AUC analysis. We observed that the only gene whose AUC value was higher than 0.80 for prognosis of nodal metastases was *B2M* (AUC=0.85, 95% confidence interval (CI)=0.69–0.94; Table 4), such that low expression of *B2M* was associated with nodal metastases. A similar AUC value (0.83, 95% CI=0.67–0.93) for *B2M* was obtained when the median value was substituted for the mean (not shown). The gene with the second highest prognostic accuracy was *GPX2* (Table 4); low expression of this gene was also associated with lymph node metastases.

Using a Δ*C*_t_ of 4.5 for a threshold for marker positivity (where Δ*C*_t_=*C*_t mean 15-gene set_−*C*_t *B2M*_), we observed that the sensitivity for nodal disease detection was 85% (17 out of 20 stage III and stage IV patients were correctly classified; see Table 3), whereas the specificity was 83% (15 out of 18 stage I and stage II patients were correctly classified; see Table 3). Interestingly, all apparent ‘false positives’ in the node-negative groups were derived from stage I patients. The relevance of this finding is discussed in further detail below.

**Figure 3:**
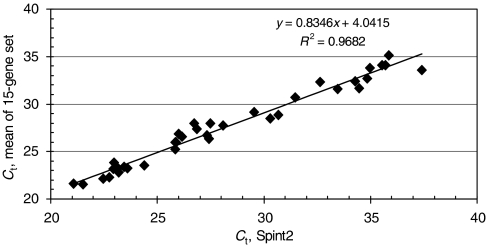
Spint2 expression levels are highly correlated with the mean of the 15-gene set. Mean *C*_t_ values of the 15 genes were obtained by dividing the sum of the *C*_t_ values by 15. In cases where no signal was detected, a *C*_t_ value of 40 was used. Linear regression analysis of the indicated data points was performed using Microsoft Excel software.

To investigate whether one or two genes from the panel could substitute for the entire set, we analysed our expression results in two manners. First, we calculated the expression ratios of all *B2M/gene X* pairs to determine whether a particular pair exhibited high prognostic accuracy. These calculations revealed that the expression ratio of *B2M*/*Spint2* had the highest prognostic accuracy of all possible pairs (AUC=0.87; 95% CI=0.71–0.96). Second, we performed a correlation coefficient analysis and determined that the mean *C*_t_ value of the 15-gene set was most highly correlated with *Spint2* (*R*^2^=0.97; Figure 3). The results of these analyses suggest that *Spint2* can be used as an internal reference control gene.

To assess the reproducibility of the *B2M/Spint2* expression ratio, the analysis described above was repeated using duplicate tissue sections and RNA that was treated with DNase. In this second analysis, we measured the expression values of *B2M*, *Spint2*, *GPX2*, *Elf3*, *CDH1*, *CDH3*, *EpCAM1*, and *CEA6*. We observed that the prognostic accuracy of the *Spint2*/*B2M* expression ratio as determined by AUC analysis was within 6% of the value observed in the first study (AUC=0.91; 95% CI=0.73–0.98; data not shown). These results provide evidence that the *B2M/Spint2* expression ratio is a reliable indicator of nodal metastases in CRC patients. Further, using the eight genes as internal reference, we observed that the prognostic accuracy of *B2M* was 0.82 (95% CI=0.63–0.94; data not shown). In a separate study conducted with a small set of tissues (*n*=14), we observed that *B2M* maintained its high prognostic accuracy for lymph node metastases (AUC=0.79) when more classical reference control genes *TBP* (Ohl *et al*, 2006) or *UBP* (Andersen *et al*, 2004) were substituted for *Spint2* (not shown).

## DISCUSSION

In this study, we observed that low expression of *B2M* was a strong prognostic indicator of lymph node metastases regardless of whether the mean expression value of the 15-gene set was used as an internal reference control (AUC=0.85; Table 4), the median expression value of the 15-gene set was used (AUC=0.83), *Spint2* was used as a single internal reference gene (AUC=0.87), or classic reference genes such as *TBP* or *UBP* were used (AUC=0.79). Based on the results described in this study, the mean (±s.d.) accuracy of *B2M* for prognosis of lymph node metastases was 0.83±0.04, a value that is sufficiently high to warrant further investigation into this biomarker. Although a previous study has shown that *B2M* was the most highly downregulated gene in CRC compared to adjacent normal tissue (Bianchini *et al*, 2006), this is the first study to show a relationship between *B2M* mRNA levels and a clinical parameter related to outcome of CRC patients.

*B2M* is known as a classic IFN-responsive gene (Yan *et al*, 2004; Gottenberg *et al*, 2006; Gray *et al*, 2006; Joyce *et al*, 2007; Scherbik *et al*, 2007; Urosevic *et al*, 2007). Interferons were originally discovered as antiviral proteins that inhibit virus replication (Isaacs and Lindenmann, 1987). Upon virus infection, IFNs are induced in mammalian cells and thus mediate cellular homeostatic responses to virus infection. In addition to their antiviral properties, IFNs are involved in many other physiological processes including cell growth and proliferation, cell death, the immune response, and other cellular defence mechanisms (Sen, 2001). In colon cancer, oncogenic K-ras inhibits the expression of IFN-responsive genes through inhibition of STAT1 and STAT2 expression (Klampfer *et al*, 2003). Under normal circumstances, IFN induces phosphorylation of STAT1, which is released from the IFN*γ* receptor and forms STAT1 homodimers. The homodimers then translocate to the nucleus to activate target genes (Darnell *et al*, 1994; Ihle and Kerr, 1995) such as *B2M*.

In addition to inactivation by K-ras mutations in colon cancer, *B2M* gene expression can also be impaired by mutations in the coding and non-coding (promoter) regions. For example, in HCT-15 colon cancer cells, both *B2M* alleles are mutated, one by an 11 base-pair deletion and the other by a point mutation, resulting in the loss of HLA class 1 surface antigens (Gattoni-Celli *et al*, 1992). Loss of *B2M* mRNA expression has important implications at the protein level. B2M is a chaperone of major histocompatibility complex (MHC) class I (-like) molecules that play a central role in antigen presentation, immunoglobulin transport, and iron metabolism. In the tumour host immune response, HLA-A,B,C assembles with B2M in the endoplasmic reticulum (Momburg and Koch, 1989; Momburg *et al*, 1989). Loss of these class 1 antigens is associated with decreased histological differentiation in colon cancer (Gattoni-Celli *et al*, 1992), as well as increased malignancy in a number of neoplasms, including B-cell lymphoma (Moller *et al*, 1987) and melanoma (Paschen *et al*, 2003). Interestingly, loss of the native HLA-A,B,C/B2M complex appears to be sporadic in nature; in some cases, the loss is localised to certain portions of the tumour, whereas in others, loss of B2M is evident across the entire tumour (Momburg and Koch, 1989). As MHC class 1 antigens are required for the host to mount a tumour response, the loss of these antigens allows a tumour to escape recognition by the immune system.

Despite the known biological properties of *B2M*, this gene has been inadvertently used as an internal reference control in studies involving IFN signalling (Einav *et al*, 2005; Tamassia *et al*, 2007), as well as real-time PCR studies of colon cancer prognosis (Takeuchi *et al*, 2003). Interestingly, in the study performed by Takeuchi *et al*, the investigators examined 36 tumours and observed that the mRNA copy numbers of *B2M* in T3/T4 cases (mean 1.78 × 10^5^ copies) had a tendency to be lower than that in T1/T2 cases (mean 4.44 × 10^5^ copies; *P*=0.16), but were not highly correlated with another reference gene. Owing to the later observation, the authors correctly concluded that *B2M* should not be used as an internal reference control. However, the investigators failed to recognise that the *B2M* gene itself might serve as a prognostic marker.

In this study, we observed that *Spint2* was a reliable internal reference control gene for CRC. This result is consistent with the study of Kataoka *et al* (2000), who found no relationship between *Spint2* mRNA and tumour stages in CRC. Further, during the course of acetic acid-induced experimental colitis in an *in vivo* mouse model, *Spint1* but not *Spint2* was upregulated in the recovery phase (Itoh *et al*, 2000), a process that requires cellular regeneration. This result further supports the concept that mRNA expression levels of *Spint2* may remain constant during tumour progression in CRC.

Spint1 and Spint2 are Kunitz-type serine protease inhibitors that regulate hepatocyte growth factor (HGF) activity through inhibition of HGF activator (HGFA), matriptase and hepsin (Parr and Jiang, 2006). Matriptase, urokinase-type activator, HGFA, and hepsin are the main factors responsible for converting inactive pro-HGF into active HGF, which is mainly secreted by stromal fibroblasts. Hepatocyte growth factor is known to play a number of roles in cancer metastasis and tumour growth. Thus, because *Spint1* and *Spint2* serve to inhibit the activity of HGF, these genes have been characterised as tumour suppressors (Morris *et al*, 2005). Further, in a study of 41 ovarian cancer patients, low expression of *Spint2* was determined to be an independent factor of poor prognosis (*P*=0.013; hazard ratio, 2.30; Tanaka *et al*, 2003). Interestingly, the suppression of metastatic behaviour (e.g. cell motility) by *Spint2* can be abrogated *in vitro* by treatment with extracellular signal-regulated kinase/mitogen-activated protein kinase and phospholipase C-γ inhibitors (Morris *et al*, 2005), suggesting that the suppressive effects of this gene can be bypassed.

Of the 14 cancer-associated genes used in the current study, none proved to be prognostic for lymph node metastases at an AUC value >0.80. This was rather surprising, as several of these genes have been previously shown to be prognostic for various cancers. For example, immunohistochemical studies have shown that low expression of *E-Cadherin* is associated with poor survival in a number of cancers including pancreatic (Shimamura *et al*, 2003), thyroid (Scheumman *et al*, 1995), gall bladder (Hirata *et al*, 2006), breast (Park *et al*, 2007), lung (Nozawa *et al*, 2006), hepatic (Wu *et al*, 2006), endometrial (Scholten *et al*, 2006), and colon (Pena *et al*, 2005) cancer. Further, we were also surprised to find that *EpCAM2/TROP2* was not a prognostic factor, as overexpression of this gene has been shown to be associated with liver metastases and survival in CRC patients (Ohmachi *et al*, 2006). It is not clear why we failed to observe a prognostic role for *EpCAM2*. In contrast to all other genes used in this study, *EpCAM2* arose from retrotransposition (of *EpCAM1*) and does not contain introns. Hence, in the absence of DNase treatment, amplification of this gene is prone to genomic contamination.

A long-range goal of our research group is to develop a prognostic assay that can be used to predict what stage II colon cancer patients might benefit from adjuvant chemotherapy. In this study, we observed that 3 out of 18 patients who were node negative by pathological assessment had low levels of *B2M*. The clinical significance of this finding is not known. However, in a subsequent study performed on untreated stage II tissues obtained from a separate institute, we discovered that low *B2M* expression was an independent prognostic marker for poor patient survival (Blum *et al*, 2008). Thus, the results described here may ultimately prove to be of benefit to a significant number of CRC patients.

## Figures and Tables

**Table 1:** Primers used for real-time RT–PCR

**Gene**	**Accession number**	**Sequence 5′–3′^a^**	**Fragment length (bp)**
AGR2	NM_006408	GCAGAGCAGTTTGTCCTCCTCA	76
		GGACATACTGGCCATCAGGAGA	
B2M	NM_004048	GCCGTGTGAACCATGTGACTTT	97
		CCAAATGCGGCATCTTCAAA	
CDH1	NM_004360	CCCACCACGTACAAGGGTC	94
		CTGGGGTATTGGGGGCATC	
CDH3	NM_001793	ATCATCGTGACCGACCAGAAT	166
		GGATGGAGTAAGCAACCACCC	
CEA6	NM_002483	AGATTGCATGTCCCCTGGAA	104
		CATTGAATGGCGTGGATTCA	
CK19	NM_002276	AACGGCGAGCTAGAGGTGA	204
		TTCCGTCTCAAACTTGGTTCG	
Claudin7	NM_001307	TGGCCATCAGATTGTCACAGAC	88
		CCAGCCAATAAAGATGGCAGG	
Elf3	NM_004433	TCTTCCCCAGCGATGGTTTT	124
		TTGCTCTTCTTGCCCTCGA	
EpCAM1	NM_002354	CGCAGCTCAGGAAGAATGTG	88
		TGAAGTACACTGGCATTGACGA	
EpCAM2	NM_002353	ACCCGAGGAGAAGAGGAGTTTG	100
		GCTTCTTTCCCAGTGACAAGCA	
GPX2	NM_002083	GGACATCAGGAGAACTGTCAGA	150
		GTCCTTCAGGTAGGCGAAGAC	
MAL2	NM_052886	GTCTGCCTGGAGATTCTGTTCG	103
		TCACGGACACAAACATGACCC	
MAP7	NM_003980	AGGACAAAGAACGCCACGAA	87
		CACGACCAACGGTTATGCTTC	
S100P	NM_005980	GACGTCTTTCCCGATATTCGG	127
		CCACGGCATCCTTGTCTTTTC	
Spint2	NM_021102	GTGCCTCAAGAAATGTGCCACT	81
		ACAGAGGAATCCGCTGCATTC	

RT–PCR=reverse transcription–PCR.

a Gene-specific primer sequences used for cDNA synthesis are underlined.

**Table 2:** Diagnostic accuracies of cancer-associated genes for detection of metastatic disease in lymph nodes derived from CRC patients

**Gene**	**AUC**	**95% CI**
Map7	0.980	0.734–1.000
AGR2	0.939	0.674–0.987
MAL2	0.939	0.674–0.987
EpCAM2	0.939	0.674–0.987
EpCAM1	0.939	0.674–0.987
CEA6	0.918	0.647–0.988
CDH1	0.918	0.647–0.988
S100P	0.837	0.548–0.972
ELF3	0.837	0.548–0.972
Sprint2	0.816	0.525–0.964
Claudin7	0.816	0.525–0.964
GPX2	0.714	0.419–0.914
CDH3	0.694	0.399–0.903
CK19	0.531	0.255–0.792

AUC=area under the curve; CRC=colorectal cancer; CI=confidence interval.

**Table 3:** Patient information

**Variable**	**Categories**	**LN+**	***P*-value**	**Low expression of *B2M*^a^**	***P*-value**
Gender	Male	12/20 (60%)	0.52	14/20 (70%)	0.03
	Female	8/18 (44%)		6/18 (33%)	
Site	Right colon	11/24 (46%)	0.63	11/24 (46%)	0.63
	Left colon	2/2 (100%)		2/2 (100%)	
	Rectum	4/7 (57%)		4/7 (57%)	
	Sigmoid	2/3 (67%)		2/3 (67%)	
	Left colon+rectum	1/1 (100%)		1/1 (100%)	
	transverse	0/1 (0%)		0/1 (0%)	
					
Type	Adeno	15/30 (50%)	0.70	17/30	0.44
	Adeno, mucinous	5/8 (63%)		3/8	
					
Differentiation	Well	3/10 (30%)	0.13	6/10 (60%)	0.82
	Well to moderate	0/1 (0%)		0/1 (0%)	
	Moderate	14/24 (58%)		13/24 (54%)	
	Moderate to poor	2/2 (100%)		1/2 (50%)	
	Poor	1/1 (100%)		0/1 (0%)	
	Continuous		0.03		0.52
					
T-stage	1	0/2 (0%)	0.001	0/2 (0%)	0.20
	2	2/12 (17%)		5/12 (42%)	
	3	17/23 (74%)		14/23 (61%)	
	4	1/1 (100%)		1/1 (100%)	
	Continuous		<0.001		0.05
					
Path stage	I	0/12 (0%)	<0.001	3/12 (25%)	<0.001
	IIA	0/6 (0%)		0/6 (0%)	
	IIIA	5/5 (100%)		3/5 (60%)	
	IIIB	2/2 (100%)		2/2 (100%)	
	IIIC	7/7 (100%)		6/7 (86%)	
	IV	6/6 (100%)		6/6 (100%)	

LN+=lymph node positive.

a Low *B2M*-expressing sample was defined by *C*_t mean 15-gene set_−*C*_t *B2M*_>4.5.

**Table 4:** Prognostic accuracies of single genes for lymph node metastases using the 15-gene set as an internal reference

**Gene**	**AUC^a^**	**95% CI**
B2M	0.846	0.692–0.942
GPX2	0.792	0.629–0.906
Claudin7	0.717	0.547–0.850
S100P	0.669	0.498–0.813
AGR2	0.665	0.494–0.810
Map7	0.653	0.481–0.799
EpCAM1	0.650	0.478–0.797
CK19	0.647	0.476–0.795
CDH1	0.632	0.460–0.782
CDH3	0.606	0.434–0.760
EpCAM2	0.601	0.430–0.756
MAL2	0.565	0.395–0.725
ELF3	0.565	0.395–0.725
Sprint2	0.546	0.377–0.708
CEA6	0.528	0.360–0.691

AUC=area under the curve; CI=confidence interval.

a Area under the curve values were obtained using the mean *C*_t_ value of the patient sample as an internal reference.
